# The Role of Teachers in Addressing Childhood Obesity: A School-Based Approach

**DOI:** 10.3390/nu15183981

**Published:** 2023-09-14

**Authors:** Anastasia Snelling, Melissa Hawkins, Robin McClave, Sarah Irvine Belson

**Affiliations:** 1 Department of Health Studies, College of Arts & Sciences, American University, Washington, DC 20016, USA; mhawkins@american.edu (M.H.); mcclave@american.edu (R.M.); 2 School of Education, American University, Washington, DC 20016, USA; sirvine@american.edu

**Keywords:** childhood obesity, teachers, nutrition education, professional development

## Abstract

Childhood obesity is one of the most prevalent public health challenges in the United States, and although rates are declining overall, rates among children living in underserved neighborhoods are increasing. This five-year intervention project seeks to empower teachers (*n* = 92) to invest in their own health and then integrate nutrition concepts into core subjects’ lessons in elementary schools. The professional development sessions reflect the concepts in the Whole Child, Whole School, Whole Community model. Results indicate that teachers who attended professional development sessions were more likely to implement nutrition lessons in the classroom (r = 0.54, *p* < 0.01), and students demonstrated a significant increase in nutrition knowledge (*p* < 0.001, df = 2, F = 9.66). Investing in school-based programs that ensure teacher well-being and professional development can yield positive benefits for both teachers and students.

## 1. Introduction

Childhood obesity has been cited as an ongoing epidemic and one of the most prevalent public health challenges in the United States [1]. In the 1970s, rates of childhood obesity were 5%; by 2008, the United States had reached a rate of 17% [2], more than tripling the prevalence in 40 years [3]. Currently, the overall rates of childhood obesity are rising at a slower rate; however, rates of childhood obesity in underserved neighborhoods are increasing at a faster pace [4]. To respond to this concerning trend, resources from federal, state, and local health agencies, along with non-profit health organizations, have focused on a myriad of solutions with schools at the epicenter, since that is where children spend much of their day and consume breakfast, lunch, and snacks.

School districts have implemented school-wide wellness policies designed to improve school meals to meet the national regulations of the Healthy Hunger-Free Kids Act [5], including garden-to-cafeteria programs. Changes in policy, systems, and environments may result in declining obesity rates [6,7,8]; however, often left out of this approach are the permanent residents of the schools—teachers. Teachers know that healthy students are better learners [9]. Including teachers in identifying multi-component solutions and integrating them into the program delivery to address childhood obesity and support child health broadly can be advantageous. Authentic partnerships with educators and schools that center their voices and perspectives as the stakeholders who spend the most time engaging with the school community who will be served are essential to this collaborative approach [10,11].

Teachers impact the lives of the students they teach. Unfortunately, due in part to the nature of the demands of schools and classrooms, it is a challenge to prioritize the health of these professionals. The COVID-19 pandemic reinforced the urgency for school districts to attend to the health and well-being of teachers, as 55% of teachers report thinking about or planning to leave the profession [12]. A recent 2022 RAND report found that nearly twice as many teachers reported job-related stress compared with the general population. The researchers also concluded that employer-provided wellness programming was associated with lower levels of job-related stress for teachers [13]. Teacher well-being can benefit students. Teachers who are healthy are better able to engage in positive teacher–student relationships [14]. Previous research has found that strong teacher–student relationships may increase attendance and engagement [15], enhance student well-being [16], reduce behavior problems [17], and contribute to positive academic outcomes [18,19,20,21].

Any new initiative, whether a program or policy, requires teachers’ buy-in for the action to reach its intended effect. Thus, teachers who are equipped and confident in translating knowledge into daily lessons play an essential role in advancing childhood obesity prevention efforts and promoting healthy lifestyles. A multi-component approach that centers on teachers’ health and well-being is a starting place to address childhood obesity at the school level. By engaging teachers with health-promoting activities and supporting healthy habits as part of their school day, teachers have an opportunity to reinforce self-care and become role models to their students [22]. In so doing, teachers are well positioned to transfer the knowledge and skills of personal health and well-being to the students in their classrooms, paving the way for this information to be applied to their lives to benefit them across their lifespans.

Teacher well-being can be defined by a range of objective and subjective psychological, physical, and social factors that encompass health, resilience, and self-efficacy [23]. Interventions to improve teacher well-being include programming to support mindfulness [24], engagement, and social connections [25]. Previous studies have found that when teachers engage in a commitment to their own health (physical and mental), students benefit from formal and informal instruction [26,27]. These positive outcomes move beyond academic productivity toward a holistic approach to both student well-being and professional autonomy for educators.

## 2. Materials and Methods

In 2010, the City Council of Washington, DC, took a bold step and passed the Healthy Schools Act (most recently amended by the Healthy Students Amendment Act 2018). This Act established comprehensive requirements to improve school meal and nutrition standards in the cafeteria and vending machines, increase student physical activity and health education time, support school gardens and farm-to-school education, and establish local school wellness policies to specifically address obesity and hunger [28]. These changes created an opportunity for the implementation of a teacher-led program in elementary schools to address childhood obesity.

Healthy Schoolhouse 2.0 is a five-year multi-component intervention funded by USDA’s National Institute on Food and Agriculture that began in 2017. The goal of this quasi-experimental prospective study is to engage teachers to teach nutrition literacy skills and knowledge to prevent obesity among elementary school students in Washington, DC. In developing a multi-level equity-focused approach, the program team utilized both the Social Ecological Model [29] and Kumanyika’s Getting to Equity (GTE) in the obesity prevention framework [30] to improve student and teacher health. Healthy Schoolhouse 2.0 aligns with the Whole School, Whole Community, Whole Child (WSCC) [31] model for introducing healthy behaviors during childhood [17] and the established correlation that healthy students are better learners [32,33]. The study design and student nutrition literacy instrument validation have been described previously [34,35]. Approval for this study was granted by the University’s Institutional Review Board in July 2017. Written informed consent was provided by teachers and students prior to data collection activities.

### 2.1. Priority Audience

One hundred percent of students in the two intervention and two comparison elementary schools reside in economically disadvantaged neighborhoods and are eligible for free breakfast and lunch. School enrollment ranged from 300 to 500 students in grades 1–5. Students attending the schools are 90–98% Black and 2–10% Hispanic/Latino. Across all four schools, 2018–2019 standardized tests show that 10–24% of students tested met or exceeded expectations in Math, and 8–12% of students tested met or exceeded expectations in English Language Arts.

Surveys were administered to participating teachers at both the intervention and comparison schools two times at baseline (*n* = 92) and post-intervention (*n* = 92). This allowed for analysis of the effect of the professional development sessions. The Teacher Health Survey (THS) includes questions regarding personal health habits, mental health, job stress, beliefs about health and education, and self-efficacy. Responses are recorded on a Likert scale from negative (1) to positive (5) to self-report agreement/disagreement regarding each item. Teachers also provided demographic information, including their gender, age, race/ethnicity, and teaching role (e.g., general classroom teachers, special education teachers). An aggregate health score was computed (range of 0–100), which included the sum of several variables: self-reported overall health, chronic condition (diabetes, asthma, and/or high blood pressure), health education beliefs (8 items), and self-efficacy (5 items). The THS has been previously administered in a similar setting and demonstrates good psychometric properties [36].

### 2.2. Research Design

The primary research arm of this study was to examine the effects of the professional development sessions in equipping teachers with the skills and knowledge to incorporate nutrition into their classroom lessons. The overarching goal was to empower teachers with strategies for integrating health into their own lives and in the classroom curriculum. The program sessions were designed collaboratively with teachers in the intervention schools. Each of the five professional development sessions started with a teacher well-being component selected by the teachers, such as healthful eating, mindfulness, or a physical activity break. This simple start was intentional and acknowledged that teachers’ well-being is important and valued. After the wellness activity, the project team then presented a sample lesson from the USDA’s *Serving up MyPlate: A Yummy Curriculum.* This curriculum was selected because it aligns with core subject standards, and therefore, teachers could incorporate food and nutrition knowledge while implementing a math, science, or English language lesson. At the intervention schools, the school principal invited the teachers to attend the sessions, and this school leader also attended and demonstrated leadership support for the wellness program. Each teacher was also provided with a complete kit that included paper copies of lesson materials, information from the professional development modules, and all other necessary supplies, such as crayons, pencils, scissors, and stickers to deliver lessons. Baseline and post-surveys were administered to students to assess nutrition knowledge and attitudes; see also [37,38].

### 2.3. Lesson Implementation

Participating teachers in intervention schools were asked to implement a minimum of three nutrition lessons over the course of the school year and to log the implementation of each lesson in an online Qualtrics (Qualtrics, Provo, UT, USA) form. Teachers could also provide feedback on the lessons and curriculum in the form. Student knowledge was assessed via the validated Student Nutrition Literacy Survey consisting of 18 items assessing nutrition knowledge, attitudes, beliefs, and intent [29].

## 3. Results

Analyses were conducted with SPSS Version 29 (IBM Corp, Armonk, NY, USA) and *R Core Team (2021) tidyverse package* (R Foundational for Statistical Computing, Vienna, Austria) [30]. Descriptive analysis was conducted to summarize teacher and student demographic characteristics. Ninety-two teachers representing both the intervention and comparison schools completed the THS pre- and post-surveys. Among teachers, demographic assessments were similar between the intervention and comparison schools at baseline. The majority of teachers were female (84.8%), identified as Black or African-American (68.5%), with a mean age of 36 years (Table 1), which is demographically representative of teachers in the Washington DC region. A total of 38% of teachers had been teaching for less than five years, and 62% had been teaching for five years or more. At baseline assessment, there were no differences among teacher average cumulative health scores by length of teaching time, grade taught, age, or gender. Aggregate health scores were 66% on average (range 34–87%) at baseline.

Fifty-five teachers in the intervention schools participated in professional development sessions (average 4 sessions, range 1–5, SD= 1.5). A total of 71 nutrition lessons were implemented by teachers in their classrooms (average 4 lessons, range 1–9 lessons). Participation in professional development sessions was associated with implementing nutrition lessons (r = 0.54, *p* < 0.01, *n* = 55) [28].

The full zero-inflated Poisson regression analysis demonstrated that self-efficacy, job stress, and professional development attendance predicted nutrition lesson implementation (Table 2). We found the full regression model fit the data significantly better by comparing it with a null model using a chi-squared test on the difference of log-likelihoods (df = 5, *p* < 0.001). Each additional professional development session attended was associated with a 48% increase in the likelihood of implementing nutrition lessons in the classroom. Each additional increase in self-efficacy score was associated with a 25% increase in the likelihood of implementing nutrition lessons. There is an inverse relationship between stress and self-efficacy; teachers who reported high levels of stress in the post-intervention THS survey scored negatively on self-efficacy items.

There was also a significant association between stress levels, cumulative health scores, and lesson implementation. Of note, over 80% of teachers reported feeling ‘very high’ or ‘high’ stress in general, which was consistent across age and gender groups. Grade, teaching role, and school type (intervention or control) were not associated with self-reported stress levels. However, older age was associated with lower stress (r = −0.24; *p* < 0.001). Further, stress scores were inversely correlated with lesson implementation (r = 0.46; *p* < 0.01); teachers who reported lower stress were significantly more likely to implement nutrition lessons than teachers who reported high stress (*p* < 0.01). Teachers who attended 1 or more professional development sessions reported lower stress scores (mean score 2.2) compared to teachers who did not attend professional development sessions (mean score 3.8) (t = 2.41, df = 17, *p* < 0.05). Teachers who did not implement nutrition lessons had an average overall health score of 63, whereas teachers who implemented 3 or more lessons had an average overall health score of 86. Previous research has demonstrated that teachers who work in urban settings consistently report higher stress than those in rural or suburban settings [39].

Demographic characteristics were similar between students in the intervention (*n* = 694) and comparison schools (*n* = 608) at baseline assessments (*n* = 1302). Participation was balanced by gender (51% male) and by grade level/age (Table 3).

Students who received lessons from teachers who participated in the professional development program advanced their nutrition knowledge. Participating students in grades 1–5 completed the brief student nutrition literacy survey at baseline and post-assessment. There were no significant differences between student pre-test knowledge scores between intervention and comparison schools. There was a significant increase in student knowledge scores among students in the intervention schools (*p* = < 0.01, *n* = 659) compared to students in the comparison schools. Students who received the intervention (3 or more lessons) had nutrition knowledge scores that were, on average, 10% higher than students with fewer lessons (0–2 lessons received) (*p* < 0.001, df = 2, F = 9.66) in post-assessments. Students who received more than 3 lessons had scores that were 8% higher on average than those who received fewer lessons (0–2) (*p* < 0.01) [40].

### Limitations

There are important limitations to consider. The professional development program was opt-in for teachers in the intervention schools, introducing a healthy worker selection bias, and not all participating teachers completed the THS at each assessment time. Both teachers and students may have received nutrition and health information from other sources that were not assessed. Although the characteristics of teachers and students were comparable at baseline, there may have been other factors in the school and home environment that contributed to differences in student knowledge and teacher health at post-intervention assessment. There was minimal sociodemographic information collected from students given the self-report assessments, which limited the ability to stratify the student survey data. Although the prevalence of childhood overweight and obesity is high in this district, clinical assessments of student body mass index (BMI) were not measured directly. This study did not assess student nutrition consumption behaviors directly; nutrition attitudes and knowledge were assessed by self-report. Generalizability is limited by the small sample size (*n* = 92) and number of schools (*n* = 4). Further, the urban geographic region is not generalizable to other school districts.

## 4. Discussion

The preliminary findings emphasize the beneficial role of professional development, health beliefs, and teacher self-efficacy. These results indicate that a short-term professional development program supporting teacher health and integrating nutrition education into core classroom subjects demonstrates the feasibility and potential for sustainability. The role of education in supporting behavior change, both for teachers and students, is underscored in the preliminary findings from Healthy Schoolhouse 2.0. The first step in behavior change is increased knowledge [41], which was demonstrated in the study results. This study sought to test the effects of a teacher-led childhood obesity program in the school setting. Unique to this study was how teachers were engaged and empowered through the professional development program. It is important to note, however, that improvements in knowledge and attitudes toward healthful eating do not necessarily translate to changes in behavior. For example, lack of access to fruits and vegetables contributes to challenges in engaging in healthy behaviors. Appropriate knowledge and skills to engage in healthy behaviors may raise confidence in self-efficacy, which may lead to changed behavior [42].

As collaborative champions, teachers best know their students, school culture, and community strengths. They are the foundation upon which school health is built and are the front door that welcomes students to develop healthy habits. Investing in teacher well-being is a short- and long-term investment in creating a culture of health and learning. Further, as the leaders of their classrooms, teachers contribute to creating a culture of health in their classrooms that may influence the larger school environment and the surrounding community. Investing in teachers’ knowledge of nutrition education is a sustainable approach to support both teacher and student well-being. Teachers are integral within the school system. Any program meant to advance student health that bypasses teachers reduces the likelihood of long-term success and scale [43].

These findings are consistent with the principles of the social-ecological model, which emphasizes the multiple spheres of influence that are mutually reinforcing; in this intervention study, the impact of teachers’ knowledge, engagement with professional development, and implementation of nutrition lessons equip teachers to be agents of change. Further, the results from this study are consistent with previous research demonstrating that teachers report high levels of job stress, which impacts their personal and physical health and well-being [44]. Papay et al. (2017) note that supportive peer relationships are protective against teacher job stress. This builds on previous research that posits that teachers have a critical role in student motivation and engagement [40] and in reducing the gap in school readiness and achievement [45]. For example, school-based prevention efforts may reduce barriers to healthful eating and support the implementation of healthy lifestyles [45,46]. In a six-month study where teachers were trained to deliver nutrition information, Rosario et al. (2012) [43] found a reduction in overweight among the students in the intervention group in grades 1–4.

To be effective, efforts to advance student health must begin with strategies to support teacher health. Professional development that addresses teacher health concerns in the workplace must be prioritized. For these opportunities to be meaningful, they must be responsive and prioritize the unique needs of each school’s teachers. Thus, regular assessments of teacher health beliefs, attitudes, and knowledge are essential to understand changing needs. These assessments can then inform professional development offerings that are tailored to and co-created by teachers themselves. Explicit investment in teacher well-being is an investment in the whole school’s health.

From a collaborative research standpoint, partnering with teachers builds capacity through empowerment while simultaneously supporting the social and economic resources of a community, two of the four key quadrants in Kumanyika’s GTE framework. Equipping teachers with the information, skills, and resources to manage their personal health allows them to become both the medium and the message in their classrooms. This poises teachers to be agents of change who know the health needs, abilities, and opportunities of their students.

Engaging teachers as partners in the prevention of childhood obesity operationalizes efforts toward achieving health equity. Policies that require teacher well-being programs to be implemented may be a first step to advancing the Whole Child, Whole School, Whole Community framework. Further, professional development opportunities for teachers designed collaboratively with teachers can translate policy mandates into effective strategies in the classroom.

### Implications for Future Research

The holistic approach described in the Whole School, Whole Community, Whole Child Model illustrates a model for creating a culture of health in the school for teachers, staff, and students [30]. Teacher engagement is a vital component to support efforts to reduce the prevalence of childhood obesity. The Healthy Schoolhouse 2.0 program centers teachers in a leadership role to support nutrition education for both teachers and students. The preliminary results of Healthy Schoolhouse 2.0 further support the feasibility of professional development sessions on health-related content as a promising strategy to support both teacher well-being and obesity prevention efforts [47].

## Figures and Tables

**Table 1:** Teacher baseline demographic characteristics.

	Comparison (*n* = 47) *n* (%)	Intervention (*n* = 45) *n* (%)	Total (*n* = 92) *n* (%)
**Gender**			
Female	40 (85.1%)	38 (84.4%)	78 (84.8%)
Male	7 (14.9%)	7 (15.6%)	14 (15.2%)
**Race**			
Black	37 (78.7%)	26 (56.8%)	63 (68.5%)
White	3 (6.4%)	13 (28.9%)	16 (17.4%)
Other	7 (14.9%)	6 (13.3%)	13 (14.1%)
**Age**			
20–29	12 (25.5%)	19 (42.2%)	31 (33.7%)
30–39	16 (34.0%)	14 (31.1%)	30 (32.6%)
40–49	11 (23.4%)	9 (20.0%)	20 (21.7%)
50–70	8 (17.0%)	3 (6.7%)	11 (11.9%)

**Table 2:** Zero-inflated Poisson regression coefficients.

Independent Variables	Estimate *	SE *	Statistic *	*p* Value	CI *
(Intercept)	−0.775	0.464	−1.671	0.095	−1.68, 0.13
Self-efficacy	0.226 (25.4%)	0.095	2.381	0.017	0.04, 0.41
Health beliefs	−0.456 (−36.62)	0.145	−3.156	0.002	−0.74, −0.17
Job stress	0.469 (59.8%)	0.177	2.640	0.008	0.12, 0.82
PD sessions	−0.654 (48%)	0.197	−3.322	0.001	−1.04, −0.27

* Estimate = regression coefficient (standardized); SE = Standard Error; Statistic = test statistic z is the ratio of regression coefficient to the standard error; CI = 95% Confidence Interval.

**Table 3:** Student baseline sociodemographic characteristics (*n* = 1302).

	Intervention (*n*= 694) n (%)	Comparison (*n* = 608) n (%)	Total (*n* = 1302) n (%)
**Gender**			
Female	329 (47.4%)	274 (45.0%)	603 (46.3%)
Male	339 (48.8%)	324 (53.2%)	663 (50.9%)
Not Reported	26 (3.7%)	10 (1.6%)	36 (2.3%)
**Grade (age)**			
1st (6–7 years)	197(28.3%)	159 (26.2%)	356 (27.3%)
2nd (7–8 years)	135 (19.5%)	111 (18.3%)	246 (18.9%)
3rd (8–9 years)	114 (16.4%)	114 (18.8%)	228 (17.5%)
4th (9–10 years)	129 (18.6%)	116 (19.1%)	245 (18.8%)
5th (10–11 years)	119 (17.1%)	108 (17.8%)	227 (17.4%)

## Data Availability

Healthy Schoolhouse 2.0 data are available upon request. The data presented in this study are available by request from the corresponding author.
